# What Has Changed in the Treatment of Psoriatic Arthritis After COVID-19?

**DOI:** 10.5152/eurasianjmed.2021.20222

**Published:** 2021-06

**Authors:** Yaşar Keskin, Gökhan Koz, Kemal Nas

**Affiliations:** 1 Department of Physical Medicine and Rehabilitation, Bezmiâlem Foundation University, İstanbul, Turkey; 2 Department of Physical Medicine and Rehabilitation, Division of Rheumatology and Immunology, Sakarya University School of Medicine, Sakarya, Turkey

**Keywords:** Psoriatic Arthritis, COVID-19, SARS-CoV-2 Virus, Treatment

## Abstract

Coronavirus disease 2019 is a respiratory infection caused by severe acute respiratory syndrome coronavirus 2. Coronavirus disease 2019 leads to the rapid activation of innate immune cells, particularly in patients with severe disease. Psoriatic arthritis is a heterogeneous chronic inflammatory disease characterized by the association of psoriasis and arthritis. Similar to those with other viruses, patients with psoriatic arthritis are at a significant risk of infection with severe acute respiratory syndrome coronavirus 2. Patients with psoriatic arthritis are immunosuppressed owing to immune dysregulation during the active disease period or owing to immunosuppressive drugs administered during remission, and they are prone to infections. The severe acute respiratory syndrome coronavirus 2 is a threat to millions of people globally owing to the decline in immunity and because a significant number of people develop severe illness. In the period of coronavirus disease 2019 pandemic, we briefly present recommendations for the treatment of psoriatic arthritis. In this review, we briefly address the management options and treatment recommendations for patients with psoriatic arthritis during and after the coronavirus disease 2019 pandemic in light of recent scientific publications.

## Introduction

Coronavirus (CoV) disease 2019 (COVID-19), which first emerged in Wuhan city of China in December 2019, is a respiratory infection caused by severe acute respiratory syndrome (SARS) CoV 2 (SARS-CoV-2), a new member of the family of CoVs.^1^ COVID-19 pandemic caused by the SARS-CoV-2 continues to have a widespread impact globally. It is life threatening owing to its highly contagious characteristics and its high risk of mortality in those with advanced age and comorbidities. In this disease with a broad spectrum of symptoms, 1 of 7 affected patients present with joint complaints (arthralgia/arthritis).^2^ Rheumatic diseases characterized by intrinsic immune dysfunction that leads to uncontrolled inflammation also pose a risk for COVID-19. The immunosuppressive properties of the drugs used in the treatment of both immune dysfunction and rheumatic diseases complicate the management of rheumatic diseases during the outbreak. The treatment of COVID-19 is mainly supportive, and approaches have been adopted for addressing the prevention/treatment of secondary complications^3^ (Table 1).

## Clinical and Research Consequences

### Current Management of Patients with Psoriatic Arthritis

Psoriatic arthritis (PsA) is a chronic disease characterized by axial involvement, peripheral arthritis, enthesitis, dactylitis, and skin, which can be heterogeneous and potentially cause severe disability. Currently, PsA treatment is based on the recommendations of the European League Against Rheumatism, Group for Research and Assessment of Psoriasis and Psoriatic Arthritis, and American College of Rheumatology. In the current practice, nonsteroidal anti-inflammatory drugs (NSAIDs), corticosteroids (limited), methotrexate, cyclosporine (less often), anticytokine biologics (anti-TNF-α, anti-interleukin [IL]-17, and anti-IL-12 and anti-IL-23), and small molecules (phosphodiesterase-4 inhibitor, Janus kinase [JAK] inhibitors) are used in the pharmacological treatment of PsA.

COVID-19, caused by the SARS-CoV-2, leads to the rapid activation of innate immune cells, particularly in patients with the severe disease.^4^,^5^ The number of circulating neutrophils is consistently higher in patients with COVID-19 who survived than in those who did not survive. Besides, this infection is associated with lymphocytopenia that mostly influences the subset of CD4+ T cells.^6^

### Clinical Course of Coronavirus Disease 2019 in Patients with Psoriatic Arthritis

The levels of several proinflammatory effector cytokines and chemokines increase in patients with COVID-19. In critically ill patients, the levels of these cytokines and chemokines rise even higher.^7^ The differentiation and activation of T cells play an essential role in COVID-19 infection. There is an important increase in the secretion of cytokines (such as IL-17) from different T-cell subtypes. Indeed, increased levels of IL-17 and TNF-α have been found in the blood (serum) of individuals with COVID-19 (especially in those who need intensive care). Studies on the use of ixekizumab for patients with COVID-19 having severe lung disease are ongoing in China.^8^

IL-17 is involved in the immunopathogenesis of PsA. Therefore, high levels of IL-17 may activate the disease. SARS-CoV-2 infection triggers a severe cytokine response in the host, which includes several mediators that are targeted in immune-mediated inflammatory diseases (IMIDs). In some patients with COVID-19, a cytokine storm resembling secondary hemophagocytic lymphohistiocytosis (HLH), a hyperinflammatory condition triggered by viral infections, develops.^9^

Similar to those with other viruses, patients with PsA are at significant risk of infection with SARS-CoV-2. Patients with PsA are immunosuppressed owing to immune dysregulation during the active disease period or immunosuppressive drugs administered during remission, and they are prone to infections. Besides, patients with PsA have an increased risk of diabetes mellitus, hypertension, and coronary artery disease owing to increased risk of metabolic syndrome compared with those with other rheumatic diseases. Frequent accompaniment of PsA with these diseases may contribute to the severe course of COVID-19.^10^ Environmental factors such as air pollution and exposure to cigarette smoke also increase the risk of severe disease.^11^

In general, considering the autoimmune and autoinflammatory rheumatic diseases, there is a severe risk of infection in those with PsA owing to organ involvement, immunosuppressive drugs and immunomodulatory therapies used, and possible comorbidities. To reduce transmission, rheumatologists should establish a remote counseling system, which will minimize contact.^12^

Some investigators have suggested that the treatment of patients with PsA should be reconsidered because staying outdoor and visits to hospitals pose a risk for SARS-CoV-2 infection. Even if there is an increased risk of COVID-19 with the immunosuppressant treatments administered in PsA as that with other rheumatic disorders, unjustified discontinuation of the immunosuppressants may have more harmful results in patients than the continuation of the treatment.^10^ Treatment of Patients with Psoriatic Arthritis During and After the Coronavirus Disease 2019 Pandemic

### General Precautions

For patients with active PsA, crowded working environment, staying outdoor, close contact, working in a hospital, obesity, advanced age, and uncontrolled comorbid disease pose a high risk. Therefore, treatment should be individualized for patients by considering clinical and social factors. It is more important not to interfere with PsA treatment when the risk of infection from the social environment is minimal and in the absence of additional comorbidity. As with other infectious diseases, biologic disease-modifying antirheumatic drug (DMARD) (bDMARD) therapy should be discontinued in patients with symptoms of SARS-CoV-2 infection. After recovery from COVID-19, the continuation of bDMARD treatment should be decided according to the medications previously used by the patients and the severity of the disease. Hyperactivation of the immune system as a result of COVID-19 infection can cause damage to the lungs and other systemic organs. During this period, immunosuppressants are also used as systemic fire extinguishers (eliminating inflammation). In addition, positive results have been reported in some recent case reports.^13^–^15^ In addition, smoking can increase the expression of angiotensin-converting enzyme 2, which plays a crucial role in the pathogenesis of COVID-19. In addition, environmental factors such as air pollution and climate change may increase the risk of COVID-19. Hence, patients should be informed of these factors because controlling them may be necessary in the course of the disease.

### Nonsteroidal Anti-Inflammatory Drugs

In some clinical manifestations of PsA (arthritis, dactylitis, enthesitis, and axial involvement), NSAIDs are used. From another point of view, the use of both NSAIDs and acetaminophen in patients may mask the symptoms of COVID-19 (such as joint and muscle pain and fever), causing a delay in the diagnosis and unfavorable management of the infection.^11^

In general, in observational studies, long-term use of NSAIDs was found to be associated with cardiovascular outcomes, including myocardial infarction, heart failure, and stroke, despite the ongoing controversy. Acute respiratory infections are already associated with an increased risk of stroke and myocardial infarction, and short-term use of NSAIDs during the disease leads to a further increase in this risk. NSAIDs cause more significant nephrotoxicity among patient groups with the likelihood of being seriously affected by COVID-19, high fever, and dehydration. Nevertheless, it is not known whether any of these pieces of evidence is valid for the COVID-19 outbreak.^16^

Oral or injectable steroids are essential and useful therapeutic agents used in the treatment of musculoskeletal system problems and rheumatological diseases. However, there are several reports stating that NSAIDs and corticosteroids can aggravate symptoms in patients with COVID-19.^17^ However, corticosteroids were used to suppress inflammation in cases of lung involvement, cytokine storm, and secondary HLH, and improvements were observed.^11^ Although there seems to be some evidence that the use of corticosteroids in the acute stage of infection may be beneficial, there is contradictory evidence from the World Health Organization regarding corticosteroid use, particularly in viral infections, which means that this evidence is not conclusive.^17^

### Corticosteroids

Current international community recommends that corticosteroid therapy should not be discontinued in patients with rheumatic disease who receive corticosteroid therapy but should be used at the lowest dose possible.^11^ In rheumatology practice, long-acting, usually insoluble steroid formulations are frequently used. For example, triamcinolone acetonide (at a dose of 40 mg) is equivalent to 10 times the normal daily physiological steroid production. It has been shown that steroids in the form of injections cause varying degrees of adrenal suppression for at least a few weeks. Intra-articular injections should only be used in active synovitis and effusion, and the lowest clinically significant doses (maximum of 40 mg depomedrone/triamcinolone for large joints and 20 mg depomedrone/triamcinolone for small joints) should be administered.^18^ In patients with PsA, the dose of steroids should not be abruptly discontinued but should be reduced if clinically possible.

Patients with PsA who are newly diagnosed and planned for treatment should be informed about the immune system dysfunctions caused by the active disease and the resulting susceptibility to infection. Treatment should be initiated with preparations that have a short-term half-life as possible. Effective corticosteroids should be administered at the lowest dose and as soon as possible in the acute period.

### Conventional Synthetic Disease-Modifying Antirheumatic Drug

Treatment should be continued using DMARDs. According to standard protocols, discontinuation of treatment or dose reduction can be considered on the basis of the severity and characteristic of the infection during the infection periods.^11^ Hydroxychloroquine has antiviral activity against SARS-CoV2 and accelerates the viral clearance in patients with COVID-19. It has also been claimed that it accelerates recovery in COVID-19, complicated by the activation of macrophages as immunomodulators and cytokine storms.^19^,^20^

### Biologic/Targeted Synthetic Disease-Modifying Antirheumatic Drugs

Although the increased risk of infection in patients receiving biological treatment has been proven, discontinuation of immunosuppressive agents leads to exacerbations of the disease. Consequently, the clinical presentation of uncontrolled systemic inflammation and systemic polyarthritis occurs. These 2 factors can cause an infectious disease with a greater predisposition to infectious diseases and poor prognosis.^21^ Consequently, biological immunosuppressive therapies have been used to suppress the body’s excessive and uncontrolled inflammatory response to infectious disease, although it appears to be contradictory. In China, a multicenter randomized controlled trial using tocilizumab (IL-6 receptor blocker used in several rheumatic diseases) in patients with high IL-6 levels in the blood and diffuse SARS-CoV-2 pneumonia has been approved.^22^ Therefore, procedures that do not affect viral clearance but inhibit increased inflammatory host response may show beneficial impacts on COVID-19.

Biological drugs are targeted for specific cytokines and appear not to increase viral infection frequencies or lead to a more severe course of viral infection in contrast to glucocorticoids.^23^

Targeting TNF and IL-6 increases the risk of bacterial infection; however, it has a low effect on viral infections (excluding the activation of hepatitis B). In particular, no association with cytokine inhibitors has been found, although the incidence of influenza and risk of developing complications from influenza infection is higher in patients with rheumatic arthritis and Crohn’s disease.^24^

Inhibition of IL-23 and IL-4/IL-13 targeted in PsA treatment does not increase the risk of viral, bacterial, or fungal infections, whereas the inhibition of IL-17A poses a risk for Candida infections and not for viral infections.^5^

JAK inhibitors targeting JAK1 and JAK3 exhibit an increased risk of herpes zoster reactivation.^5^ Targeting of JAK1 and JAK3 affects the function of various cytokines involved in antiviral responses such as type I interferons (IFNs), IL-2, IL-15, IL-21, and IFN-y. Therefore, JAK1/JAK3 inhibitors can theoretically inhibit the clearance of SARS-CoV-2. Consequently, it was reported that JAK2 inhibition seems to block viral entry of SARS-CoV-2 and IL-17-induced cytokine activation.^25^ IL-6 and GM-CSF cytokines act partially or entirely, depending on the JAK2 signal pathway. Thus, JAK2 could be a target for treating hyperinflammatory response in COVID-19.^5^

Although patients with rheumatic diseases are particularly vulnerable to the virus, in some cases, they can be protected unexpectedly owing to the drugs used to control their disease. One of the most important causes of the critical disease in patients with COVID-19 is cytokine storming, which often leads to death.^9^,^12^,^26^

Drugs used in autoimmune diseases have anti-inflammatory properties that can protect against proinflammatory cytokines released during a cytokine storm associated with COVID-19.^27^

In a study conducted in Italy, 4 of 320 patients—of whom 53% had rheumatoid arthritis and 43% had spondyloarthritis (of these patients, 52% received TNF-α inhibitors, 40% received other bDMARDs, and 8% received targeted synthetic DMARDs [tsDMARDs])—were diagnosed with COVID-19 by rhinopharyngeal swabs. Symptoms that were very suggestive of COVID-19 were also reported in 4 other patients. All patients with confirmed COVID-19 received at least 1 antibiotic therapy, and the hospitalized patients were administered antiviral therapy and hydroxychloroquine. The bDMARDs or tsDMARDs used by all the patients with symptoms of infection were temporarily discontinued from the onset of symptoms. No significant recurrence of rheumatic disease was observed in these patients. None of the patients with a confirmed diagnosis of COVID-19 developed severe respiratory complications, and no mortality has been observed. Only a patient aged 65 years needed hospitalization for several days and was given low-flow oxygen support. No increase in mortality was reported in patients undergoing immunosuppression for organ transplantation, cancer, or autoimmune diseases during different CoV outbreaks, including during SARS and the Middle East respiratory syndrome (MERS) caused by SARS-CoV and MERS-CoV outbreaks, respectively, which belong to the same family of CoVs as SARS-CoV-2.^28^

Currently, there is limited evidence on how COVID-19 affects patients with IMIDs treated with cytokine inhibitors. However, considering the role of proinflammatory cytokines in the pathophysiology of COVID-19 and analyzing the risk of viral infection during anticytokines therapy, it is anticipated that most cytokine inhibitors used in the treatment will not put patients at risk of developing more severe COVID-19.^12^,^29^,^30^

In conclusion, it is essential to provide low disease activity in patients with PsA during the pandemic. Only in individuals diagnosed with COVID-19 or in those with high-risk COVID-19 contact or immunosuppressive, immunomodulating, or biological treatment should it be discontinued. Immunosuppressive treatments for PsA seem to increase the risk of infection; however, immunological deterioration due to drug discontinuation may lead to both exacerbation of the disease and increased risk of infection.

Hydroxychloroquine, which has long been used for the treatment of IMID, has shown efficacy in COVID-19 management.^31^ In addition to these studies, registers are being established to promote better understanding regarding the effect of COVID-19 in patients with autoimmune disease and to potentially reveal a protective role of specific cytokine-inhibition strategies.^5^

### Limitations

This study had certain limitations that need to be addressed. There was considerable confusion and uncertainty regarding the treatment strategies, especially during the early stages of the pandemic. As in our clinic, most hospitals and clinics dealt exclusively with pandemic patients, resulting in an interruption of rheumatology patients’ follow-ups. Regular record keeping was also interrupted. Furthermore, because the data on COVID-19 outcomes in patients with PsA are limited in the literature, we were able to make evaluations only on the basis of the results of the existing cases or case series. However, we could make comparisons using similar reports on infectious diseases published before the COVID-19 pandemic. Therefore, the results and recommendations we present in this paper should be interpreted taking the information mentioned earlier into consideration (Table 2).

## Conclusion

The novel CoV SARS-CoV2 is a threat to millions of people globally owing to the decline in immunity associated with it and because a significant number of people develop severe illness. Quarantine and timely diagnosis are essential for COVID-19 outbreak control. With the effects of integrated interventions, such as promoting the use of face masks and reducing travel, cumulative infected cases and deaths are estimated to decrease.^32^

Although COVID-19 has spread in a similar manner to SARS and MERS, it is known to have lower mortality rates. Owing to globalization, the CoVs of different mutant strains are expected to cause similar spreads and outbreaks in the coming years. The emergence of tissue- and stage-specific agents contributing to the pathology will result in new, useful, and disease-specific therapeutic procedures that control virus replication while limiting inflammatory damage until vaccines are available. With the increasing scientific collaboration as a result of globalization, in the future, we may have more potent fighting tools against CoVs, whose genome is very well known.^33^

## Figures and Tables

**Table 1:** Clinical Characteristics of Patients with PsA Diagnosed with COVID-19

Article	Age/sex	Type of study	Disease/ severity	Patients comorbidity	Medical history	Imaging findings	Presenting COVID-19 symptoms	COVID-19 courses and management of the disease
Messina^13^	32/F	Case report	PsA/Mild	NR	Guselkumab/ methotrexate	NR	Mild rhinorrhea, fever	Only symptomatic treatment was administered. Methotrexate and guselkumab treatments were discontinued. Her symptoms improved within 2 weeks.
Di Lernia^14^	73/F	Case report	PsA/Mild	Hypertension, tachycardia, osteoporosis, fractures, hyperuricemia	secukinumab	NR	Fever, sore throat, mild cough	Not hospitalized. HQ regimen was given for 7 days. Improvement in symptoms was observed after about a week. After 28 days, the results of two consecutive RT-PCR tests were negative. Secukinumab injection was continued during the infection
Haberman^15^	73/M	Case series Case 1	PsA/ Moderate	Diabetes, BMI>40	Tofacitinib	CXR: Diffuse patchy opacities	Fever, cough, shortness of breath	Hospitalized and received O_2_ support through nasal cannula in the general ward. HQ/azithromycin was administered. Discharged on day 14.
	51/M	Case 2	PsA/Mild	Hypertension BMI>35	Secukinumab	CXR: Prominence of bronchovascular markings	Fever, cough, shortness of breath	Hospitalized in the general ward and received O_2_ support through the nasal cannula. HQ/azithromycin was administered. Discharged on day 3.
	62/M	Case 3	PsA/Mild	Hypertension BMI>30	Methotrexate 20 mg	CXR: Diffuse patchy opacities	Fever and shortness of breath	Hospitalized and received ICU-level care in addition to ARDS ventilator assistance. HQ/azithromycin/ lopinavir-ritonavir were administered. Discharged on day 24
Favalli^29^	68/F	Case report	PsA/NR	NR	NR	NR	NR	Not hospitalized. Treatment details were not reported. The patient, who had no respiratory complications, was treated at home.
Messina^30^	53/M	Case report	PsA/Mild	Non	Ustekinumab	CXR were suggestive for SARS-CoV-2 infection.	Low-grade fever, cough, general malaise	ICU level care and dyspnea requiring noninvasive ventilation. Chloroquine, lopinavir and ritonavir, methylprednisolone, paracetamol, and azithromycin were administered. Symptoms improved after about 20 days. Ustekinumab injection was continued during the infection.

ARDS: acute respiratory distress syndrome; BMI: body mass index; COPD: chronic obstructive pulmonary disease; COVID-19: coronavirus disease 2019; CRP: C-reactive protein; CXR: chest X-ray; F: female; HQ: hydroxychloroquine; ICU: intensive care unit; IL-6: interleukin-6; M: male; NR: not reported; O_2_: oxygen; PsA: Psoriatic arthritis; RT-PCR: reverse transcription-polymerase chain reaction.

**Table 2:** Recommendations for the Management of Patients with PsA during the COVID-19 Pandemic

Medicines	Recommendations
NSAIDs	They should not be used in patients with severe organ failure and severe respiratory symptoms.
Corticosteroids	They can be used before COVID-19 exposure and during the infection. However, they should be used at the lowest possible dose and should not be discontinued abruptly.
csDMARDs	They can be used before COVID-19 exposure. However, csDMARDs other than salazopyrin and hydroxychloroquine should be discontinued in patients with suspected contact. During the infection, only hydroxychloroquine treatment can be continued, whereas other csDMARDs should be discontinued.
b/tsDMARDs	They can be used before COVID-19 exposure. If possible, intravenous forms should be replaced with subcutaneous forms, and dose intervals should be extended. Treatment should be discontinued if diagnosed with COVID-19 (continuation of the IL-6 blocker tocilizumab, which is recommended only for severe COVID-19 cases, may be considered).
Vaccination	Pneumococcal and flu vaccines are recommended for all patients.

b/tsDMARD: biologic/targeted synthetic disease-modifying antirheumatic drug; COVID-19: coronavirus disease 2019; csDMARD: conventional synthetic disease-modifying antirheumatic drug; DMARD: disease-modifying antirheumatic drug; IL-6: interleukin-6; NSAID: nonsteroidal anti-inflammatory drug.
